# Subcutaneous panniculitis-like T cell lymphoma in a patient with multiple sclerosis during anti-CD20 therapy: a case report

**DOI:** 10.1007/s00415-026-14233-x

**Published:** 2026-10-30

**Authors:** Fabio Amati, Concetta Chiapparino, Giovanni Franco, Gianluca Frisone, Bonaventura Ardito

**Affiliations:** Neurology Unit, Ospedale della Murgia “Fabio Perinei”, Altamura, Italy

Dear Sirs,

Teofilo and colleagues [1] recently described the case of a 42-year-old woman with multiple sclerosis (MS) who was treated with ofatumumab, a fully human anti-CD20 monoclonal antibody (mAb); the patient subsequently developed mycosis fungoides and switched to natalizumab for safety reasons. Here, we present a second example of a lymphoproliferative event in MS during anti-CD20 therapy.

We report the case of a 41-year-old female diagnosed with relapsing–remitting MS in January 2020, at the age of 35. She was initially treated with ocrelizumab (a humanised mAb that selectively targets CD20+ B cells) and rapidly experienced a marked reduction in serum IgM levels. This was followed by the appearance of an erythematous lesion in the inframammary fold, interpreted as hidradenitis suppurativa (August 2023), and the onset of a soft-tissue lesion involving the right shoulder (October 2024). These events, along with persistent low serum IgM concentration, led to discontinuation of ocrelizumab therapy for safety concerns (last administration in May 2024).

The patient was later diagnosed with an inflamed sebaceous cyst in the right suprascapular region with lymphocytic infiltrate (December 2024–January 2025). Its biopsy documented a chronic granulomatous giant cell phlogosis with minimal eosinophilic component. Given the patient’s low-intermediate serum anti-JCV antibody levels (STRATIFY index 0.70; ImmunoWELL index 1.08), natalizumab was chosen for maintenance treatment (May 2025). A second biopsy of the same lesion (July 2025) identified histological findings consistent with both lymphocytic panniculitis and a panniculitis-like T cell lymphoproliferative process/T cell lymphoma. Molecular analysis showed monoclonal rearrangement of T cell receptor (TCR) gamma genes, and EBV testing was negative. Based on these results, natalizumab administration was therefore stopped.

Lesions developed on the patient’s face, trunk, and legs (August 2025). Peripheral T cell lymphoma with features of subcutaneous panniculitis-like T cell lymphoma (SPTCL) was finally diagnosed histologically (September 2025).

In October 2025, whole-body FDG PET-CT showed multiple areas of increased glucose metabolism throughout the subcutaneous tissue. Whole-body CT revealed the presence of subcutaneous solid tissue at the left cheek, the right scapular region, and the left paramedian cervical and dorsal regions. Thin strands of pathological tissue were observed extending along the entire spine, including the right paramedian area. No lymphadenopathy was detected.

In November 2025, the patient started an intensive chemotherapy regimen (CHOEP – cyclophosphamide, hydroxydaunorubicin [doxorubicin], Oncovin [vincristine], etoposide, prednisone), which was completed in March 2026. Brain and spinal cord MRI showed no radiological signs of disease activity (May 2026), and no clinical relapses have occurred since discontinuation of disease-modifying therapy (DMT).

There are similarities between these reports. Both female patients were treated with anti-CD20 mAbs and developed a cutaneous lymphoproliferative event at almost the same age, leading to the interruption of the respective therapy and a switch to natalizumab. Clinical differences must also be acknowledged. In the first case, the patient had received several DMTs prior to anti-CD20 therapy, whereas our patient was DMT-naïve. The treatment duration of ofatumumab was approximately half that of ocrelizumab. Mycosis fungoides and SPTCL represent distinct primary cutaneous T cell lymphomas, each one with specific anatomical tropism, clinical presentation, and therapeutic protocol. Mycosis fungoides is an epidermotropic CD4+ T cell malignancy that typically manifests as superficial erythematous patches, plaques, and tumours, preferentially distributed across sun-shielded areas [2]. Conversely, SPTCL usually presents as painless subcutaneous nodules or deep-seated plaques resembling panniculitis, predominantly located on the extremities and trunk [3].

Sequential dynamics of ocrelizumab-induced immune modulation in MS show a biphasic effect: immediate, sustained suppression of B cell-related pathways, followed by gradual downregulation of T cell-mediated immunity [4]. Given that mycosis fungoides and SPTCL are rare subtypes of cutaneous T cell lymphoma, we could speculate that a relationship between anti-CD20 therapy and the occurrence of lymphoproliferative diseases in patients with MS exists. Although causality cannot be established, prolonged B cell depletion could plausibly perturb immune regulation and affect T cell homeostasis because B cells contribute to antigen presentation, cytokines production, and modulation of effector and regulatory T cell responses [5–7]. This hypothesis is biologically relevant since regulatory B cell populations help maintain immune tolerance by suppressing pathogenic T cell responses and supporting regulatory T cell function [8]. Interestingly, rare T cell lymphoproliferative disorders have been reported after B cell depletion induced by CD19-directed CAR T therapies, even though the absolute risk appears very low and causality remains uncertain [9–11]. Current data suggest that any such association is likely multifactorial, potentially involving immune dysfunction, viral reactivation, clonal hematopoiesis, and, rarely, vector-related events, and therefore warrant further investigation [10–13]. However, mycosis fungoides has also been described in patients with MS during treatment with fingolimod [14]. This fact limits the possibility of attributing the observed lymphomas specifically to anti-CD20 therapy.

For our patient, we chose natalizumab after ocrelizumab because of its well-established safety profile and distinct mechanism of action (inhibiting lymphocyte trafficking without B cell depletion), which, in theory, preserves peripheral immune surveillance. Indeed, a switch to natalizumab as a sequential strategy for people receiving anti-CD20 treatment may be appropriate, particularly in cases with an increased incidence of infection [15].

The onset of rare lymphoproliferative disorders in patients with MS treated with anti-CD20 mAbs cannot be considered a drug-related effect by itself. Given the lack of epidemiological evidence supporting an increased risk, a coincidental occurrence is likely. Nonetheless, these cases highlight the importance of ongoing monitoring to balance therapeutic benefits with potential adverse events.

## Data Availability

No datasets were generated or analysed during the current study.
